# Mismatch detection in homologous strand exchange amplified by hydrophobic effects

**DOI:** 10.1002/bip.23426

**Published:** 2021-03-29

**Authors:** Bengt Nordén, Tom Brown, Bobo Feng

**Affiliations:** ^1^ Department of Chemistry & Chemical Engineering Chalmers University of Technology Gothenburg Sweden; ^2^ Chemistry Research Laboratory, Department of Chemistry University of Oxford Oxford UK

**Keywords:** DNA strand exchange, mismatch detection, PEG, hydrophobic catalysis

## Abstract

In contrast to DNA replication and transcription where nucleotides are added and matched one by one, homologous recombination by DNA strand exchange tests whole sequences for complementarity, which requires elimination of mismatched yet thermodynamically stable intermediates. To understand the remarkable sequence specificity of homologous recombination, we have studied strand exchange between a 20‐mer duplex containing one single mismatch (placed at varied positions) with the matching single strand in presence of poly(ethylene glycol) representing a semi‐hydrophobic environment. A FRET‐based assay shows that rates and yields of strand exchange from mismatched to matched strands rapidly increase with semi‐hydrophobic co‐solute concentration, contrasting previously observed general strand exchange accelerating effect of ethyl glycol ethers. We argue that this effect is not caused simply by DNA melting or solvent‐induced changes of DNA conformation but is more complex involving several mechanisms. The catalytic effects, we propose, involve strand invasion facilitated by reduced duplex stability due to weakened base stacking (“longitudinal breathing”). Secondly, decreased water activity makes base‐pair hydrogen bonds stronger, increasing the relative energy penalty per mismatch. Finally, unstacked mismatched bases (gaps) are stabilized through partly intercalated hydrophobic co‐solvent molecules, assisting nucleation of strand invasion at the point of mismatch. We speculate that nature long ago discovered, and now exploits in various enzymes, that sequence recognition power of nucleic acids may be modulated in a hydrophobic environment.

## Graphical abstract

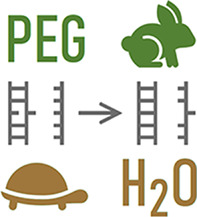

## 1 INTRODUCTION

High fidelity DNA synthesis is crucial for maintaining genetic information over many generations, and to avoid mutations that can lead to cancer or neurodegenerative disease. Cells harbor multiple DNA polymerases several only discovered recently and with functions not yet fully understood.^[^ ^1^, ^2^ ^]^ The nucleobases are responsible for the coded information but not themselves main attractors in the recognition machinery which makes high‐fidelity recognition mechanisms complex both in DNA polymerase replication, RNA polymerase transcription as well as in homologous DNA recombination. The mechanisms of recombination enzymes are similar,^[^ ^3^, ^4^ ^]^ they first bind to a single‐stranded part of DNA to form a filamentous complex with DNA which is stretched about 50% in length. This single‐stranded (ss)DNA‐RecA filament then interacts with a double‐stranded (ds)DNA to form a ssDNA‐RecA‐dsDNA complex and if the two DNAs have identical sequence, strand exchange occurs. Despite importance of recombinases in health contexts (e.g., cancer, gene therapy, sterility) and many years' intense research, the mechanisms of searching for homology and executing strand exchange are not yet understood at a molecular level and many questions, including why the DNA is stretched, remain enigmatic. An improved fundamental understanding of the mechanistic details of these processes could pave way for many important applications, such as the CRISPR technology, where incorporation of new DNA relies on the cell's native recombination machinery.^[^ ^5^, ^6^, ^7^ ^]^

There could be several explanations for absence of breakthroughs in homologous recombination research and why it appears stagnant compared to the explosive development of CRISPR‐Cas involving RNA‐DNA recognition. One is that elucidating reaction mechanisms is challenging as the system involves very long nucleoprotein filaments of many RecA molecules. Details how RecA interacts with DNA are still elusive, including roles of two dangling peptide loops where studies indicate proximity to DNA and crystal structure shows triplets of stacked bases sandwiched between L2‐hairpins with base edges solvent‐exposed.^[^ ^3^, ^4^, ^8^ ^]^ Another, more dramatic reason why recombination mechanisms have remained elusive could be that something is wrong with the basic theory of DNA interactions, which requires complete rethinking. In the RecA‐DNA context, RecA being one of our oldest well‐preserved proteins, the mentioned free peptide loops might provide a clue: hydrophobic parts of a loop could catalyze recombination either by stabilizing unstacked bases by direct interaction (e.g., partial intercalation), or indirectly by osmotic or dehydration effects. Thus, in addition to well‐defined interactions we propose indirect influence from modulated water activity and dielectric medium effects: they can affect stacking energy and reinforce hydrogen bonds from RecA to DNA phosphate oxygens (thus a sequence‐independent effect).

We recently presented evidence that certain semi‐hydrophobic co‐solutes can attenuate nucleobase stacking, leading to increased DNA flexibility, transient unstacking events and lowered activation energy to intercalation.^[^ ^9^ ^]^ Similar agents are able of catalyzing spontaneous strand exchange between homologous DNA molecules.^[^ ^10^, ^11^ ^]^ We hypothesize that bacterial RecA and eukaryote Rad51 may use similar strategies to disrupt DNA stacking and catalyze strand exchange. Base‐pair hydrogen bonds were earlier seen as the glue holding complementary DNA strands together, today it is accepted that the DNA double helix is mainly stabilized by hydrophobic and dispersive interactions between nucleobases in their coin‐pile stacked B conformation.^[^ ^12^, ^13^, ^14^, ^15^ ^]^

Potentially related to the stretched DNA in recombinase complexes, is our finding that GC‐rich DNA exposed to mechanical pulling force displays a distinct conformation (∑ DNA) almost exactly 50% longer than normal DNA.^[^ ^16^, ^17^, ^18^ ^]^ Neither the stretching nor the hydrophobic effect is associated with any significant base‐pair opening (denaturation), and both effects blatantly involve cohesive π‐stacking energy. We believe both of these physical properties inherent of the DNA structure are somehow exploited by nature in homologous recombination and repair reactions,^[^ ^19^ ^]^ catalyzing the reactions and also improving the sequence recognition fidelity as demonstrated in this communication.

## 2 EXPERIMENTAL DETAILS

**FIGURE 1:**
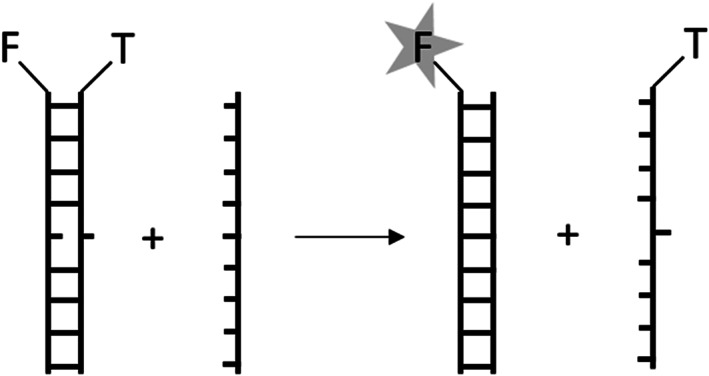
Schematics of resolving a mismatched duplex through DNA strand exchange. The FAM label of the mismatched duplex is quenched by TAMRA on the mismatched strand. Fluorescence is restored upon strand exchange with an unlabeled matching strand

Figure 1 outlines the FRET assay used to monitor DNA strand exchange. DNA was purchased from ATDBio (synthesized on the 1 μmol scale using standard solid phase protocols and HPLC purified before delivery). A FAM‐labeled and a TAMRA‐labeled strand (matching or mismatching) were annealed by cooling linearly 90‐10 °C over six hours, forming a 20‐mer DNA duplex. A duplex mismatched at position X is called mX for short (sequences in Supporting Information, Section 1). A third unlabeled strand, complementary to the FAM strand, is introduced five times in excess. Strand exchange separates the quenched FRET pair and restores FAM fluorescence, which is directly proportional to the strand exchange yield. The pseudo‐first order rate constant *k* can be obtained by fitting the equation *y* = 1 – *A*·exp(–*k*·*t*) to the normalized kinetic traces (details in Supporting Information, Section 2).

Each experiment used 2 × 10^–10^ moles of the initial duplex in a final sample volume of 1 mL. Fluorescence was measured on a Varian Eclipse fluorometer with 1 second collection time, 496 nm excitation, 518 nm emission, 5 nm slits, and 600‐800 V photomultiplier voltage to maintain an intensity below the maximal 1000. Temperature (37 °C for kinetics) was controlled using the heating block accessory. The standard buffer was sodium phosphate (prepared from mono‐ and disodium phosphate (analytical grade, Sigma‐Aldrich) and purified water (Milli‐Q) with [Na^+^] = 50 mM, pH 7.5, and additional sodium chloride (analytical grade, Sigma‐Aldrich) was added to perform *T*_m_ matching between matched and mismatched strands (details in Supporting Information, Section 4). Polydisperse PEG‐6000 (average m. w. 6000 g/mol, “Bio Ultra” grade, Sigma‐Aldrich) was dissolved under slow inversion of the flask. PEG concentration is given as weight percentages.

## 3 RESULTS

**FIGURE 2:**
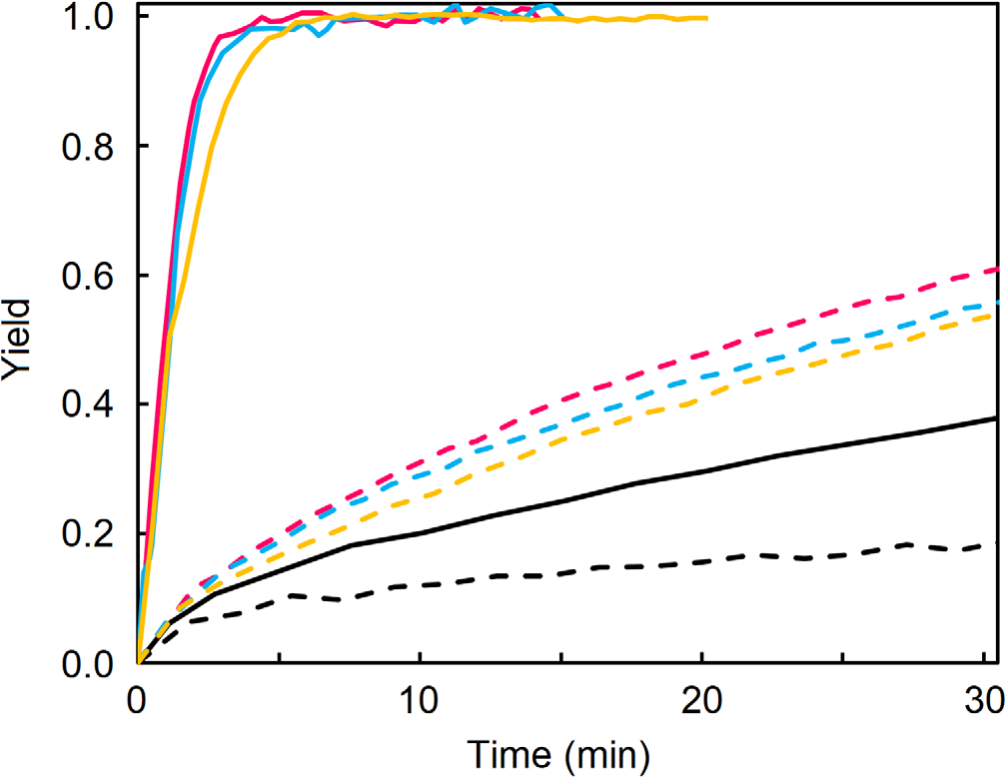
Representative strand exchange kinetic traces for matched (black) and mismatched (m4 red, m5 yellow, m10 blue) DNA in buffer (dotted) and 45% PEG (solid). While the exchange rates are approximately the same for matched and mismatched strands in buffer, they differ greatly in presence of 45% PEG

Select strand exchange kinetic traces for matched and mismatched DNA duplexes in the presence and absence of PEG are presented in Figure 2. It can be seen from the data that the hydrophobic environment created by 45% PEG is not strong enough to significantly accelerate strand exchange of the matched DNA. By way of contrast, strand exchange is greatly accelerated by one mismatched base in the presence of 45% PEG, even when compared to mismatched strand exchange in pure buffer. It is important to note that a mismatched base only slightly influences the strand exchange kinetics in the absence of PEG, as evident when comparing the colored dotted lines (mismatched) to the black dotted line (matched) in Figure 2.

Fluorescence melting curves due to DNA heat denaturation (Supporting Information, Section 4) show that the melting temperature (*T*_m_) of the DNA duplexes is suppressed by the presence of a mismatched base, by approximately 7 °C for all PEG concentrations investigated, and approximately 5 °C in absence of PEG. A trivial explanation to the enhanced sequence specificity in PEG solutions could be that PEG decreases the stability of the initial mismatched duplex disproportionately more than the stability of the matched duplex. However, a simple argument against this explanation is that *T*_m_ decreases by the same amount in all concentrations of PEG, yet the mismatched/matched ratio increases with PEG concentration.

**FIGURE 3:**
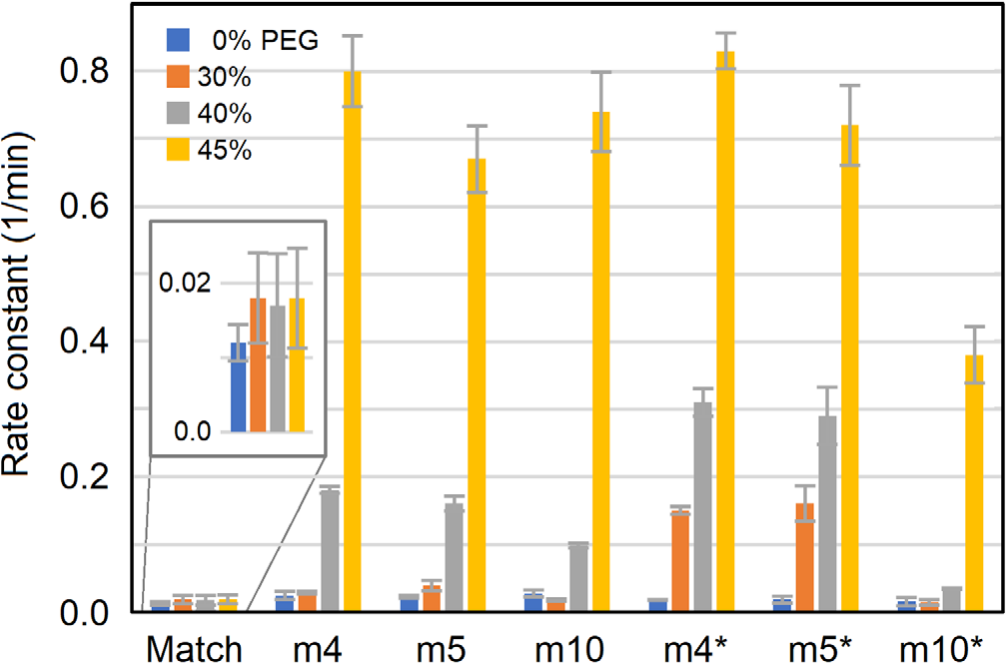
Strand exchange rate constants (min^−1^) for matched (inset) and mismatched duplexes in different PEG concentrations. An asterisk indicates that extra salt was added to increase the melting temperature. Error bars indicate ± SD (n = 2). See fitting details in Supporting Information, Section 2

In Figure 3, rate constants are presented for matched and mismatched strands in several PEG concentrations. In pure buffer, the rate constants are only slightly higher for mismatched strands compared to matched strands. However, this difference increases with PEG concentration. At 45% PEG, mismatched strands are exchanged approximately 40 times faster than the matched strands, seemingly independent from the mismatch position. To definitely exclude the trivial explanation from previous paragraph, additional salt (details in Supporting Information, Section 4) was added to increase *T*_m_ of mismatched DNA. The results are marked with an asterisk in Figure 3. For each PEG concentration, the three mismatched duplexes have at least the same *T*_m_ as the matched duplex.

It is only natural to introduce the mismatched/matched ratio between the rate constants of mismatched strand exchange and matched strand exchange, with the PEG concentration kept equal. This ratio is a fair measurement of the fidelity of nucleobase pairing, since it is derived from the process of replacing a faulty strand in a mismatched DNA duplex with a matching strand of the correct sequence. In other words, the mismatched/matched ratio, rising from approximately 2 in pure buffer to approximately 40 in 45% PEG, reflects an increased base pairing fidelity in a more hydrophobic medium.

Even after *T*_m_ adjustment, obviously, mismatched duplexes undergo strand exchange much faster than matched duplexes. The only exception is m10 exchanging more slowly with extra salt, although still significantly faster than the matched duplex which has the same melting temperature (and lower salt concentration). Thus, we dismiss the trivial explanation as highly unlikely. Unexpectedly, in some cases (m4, m5, 30%‐40% PEG), addition of salt seems to somewhat accelerate strand exchange. This is most obvious when comparing the orange staples in Figure 3. Perhaps, under certain conditions and for certain DNA sequences, DNA strand invasion is facilitated by electrostatic shielding between the charged phosphate groups on the original duplex and the approaching third strand. On the other hand, this salt effect is not seen in the absence of PEG and therefore does not accelerate strand exchange on its own. We leave this open for further studies, if more mismatched sequences could be tested, and PEG and salt concentrations varied more systematically.

It can also be seen from Figure 3 that for high salt concentrations, the position of mismatch influences the strand exchange kinetics. While the duplexes containing m4 and m5 behave quite similarly for all PEG concentrations, having their mismatch close to one end of the DNA duplex, m10 exchanges significantly more slowly than the other two sequences, both having a mismatch in the middle. We can explain this difference in a logical manner by noting that duplex breathing becomes progressively more difficult with higher salt concentration due to increased DNA duplex stability, so the dominant duplex breathing mode would be fraying at the ends rather than opening in the middle. Therefore, strand invasion in the middle of the sequence becomes much more unlikely although strand invasion at the ends is largely unaffected.

## 4 DISCUSSION

Our results indicate that a mismatched duplex is converted more readily into a matched duplex through strand exchange if non‐ionic, semi‐hydrophobic PEG is present. The mismatched/matched ratio between strand exchange rates increases markedly with PEG concentration, which means that the hydrophobic environment is important for the specificity of sequence recognition of DNA. It is known that close to melting of DNA the discrimination power is strongly enhanced, as was demonstrated by the detection of single base mutations in the cystic fibrosis gene with PNA at elevated temperature.^[^ ^20^ ^]^ Any denaturing solvent should have such a “thermal” effect, but several observations indicate that the effect of our semi‐hydrophobic co‐solute is different from non‐specific thermal activation and thermodynamic discrimination that closeness to Δ*G* = 0 implies. One conspicuous effect is due to that the reduced water activity by dilution and by presence of hydrophobic surfaces will stabilize base‐pairing hydrogen bonds making the matching base‐pairs relatively more stable than the mismatched. The base‐pair strengthening effect in a non‐polar environment was demonstrated with benzoic acid whose hydrogen‐bonded dimer, serving as model for an A‐T base‐pair with two parallel hydrogen bonds, was preferentially populated in a polyethylene matrix,^[^ ^19^, ^21^ ^]^ quantum mechanical calculations indicating a destabilization due to removal of competing water hydrogen bonding by nearly 3 kcal/mol (per dimer).

The ability of DNA to recognize its complementary sequence is an abstract concept and could be defined in several ways. In this article, we study the conversion of a mismatched DNA duplex into a matched one through exchange with a third fully complementary strand. If this conversion is somehow facilitated by some general catalytic function, then the number of mismatched bases will be decreased, so the specificity of base paring could be said to be increased. In a strict sense, firstly, strand exchange of a mismatched duplex must be facilitated. Secondly, the mismatched/matched ratio between rate constants must increase. If this second requirement is not met, then only the general rate of strand exchange has increased, but not the sequence specificity.

It is also interesting that the melting temperature does not immediately predict the rate of strand exchange. Comparing *T*_m_ in the absence of PEG, with *T*_m_ in 30% PEG, the latter is overall higher, probably due to the stabilizing effect of PEG acting as a crowding agent. However, strand exchange in 30% PEG is not generally slower than in pure buffer. Also, when considering the results obtained when adding extra salt to increase *T*_m_, it can be concluded that thermodynamic stability (expressed as *T*_m_) of DNA is at least partially separate from its availability to reactions (expressed as the rate constant *k*).

Attempts to determine activation energies have failed mainly because the estimates of rate constants are too crude (kinetics being generally multi‐exponential) and because of too limited temperature range without melting phenomena. By studying the effect of added ethylene glycol ethers to single DNA molecules subject to pulling forces, a reduction by approximately 20% in critical force has been noticed in 20% diglyme.^[^ ^9^ ^]^ In pure aqueous buffer, short DNA (60‐120 base‐pairs) was found to undergo a conformational transition at a critical force corresponding to an activation free energy of 1.6 kcal mol^‐1^ (base‐pair) which fits well theoretical estimates of pi‐stacking energy.^[^ ^13^ ^]^

Given that the rate of strand exchange depends on DNA sequence, some insight could be gained about the actual mechanism of strand exchange. Our data support that the rate limiting step would be a strand breathing event which acts as a nucleation site for strand invasion through diffusion of a third strand, rather than the formation of some temporary triple‐stranded intermediate. There would be no advantage then in having a mismatch close to the end in forming such an intermediate.

An earlier report by Westerlund *et al*., which used charged liposomes to attract DNA to accelerate strand exchange,^[^ ^22^ ^]^ showed that end fraying and mismatches close to the ends contribute more to fast strand exchange rates. Furthermore, Maruyama and co‐workers have studied highly cationic polymers which catalyze mismatched DNA strand exchange,^[^ ^23^, ^24^ ^]^ and a mismatch close to the end was found to exchange faster.^[^ ^24^ ^]^ However, because their mismatch is carried on the single strand, their forward reaction is the opposite to Figure 1. Therefore, in terms of Figure 1, the charged polymers cause a mismatch close to the end to exchange slower than a mismatch in the middle. Overall, differences between the works of Westerlund and Maruyama, and the present article, could show that using cationic charges to accelerate strand exchange may have several different mechanisms, while hydrophobic catalysis of strand exchange could have yet another explanation.

Finally, is the strand exchange accelerating effect of PEG due to hydrophobic interactions or molecular crowding? PEG‐6000 is known to exert a large volume of exclusion and therefore a strong crowding effect. In earlier papers,^[^ ^9^, ^10^, ^11^ ^]^ by using short ethylene glycol ethers (glyme, diglyme, and dioxane) we have argued that the hydrophobic effect dominates. We also introduced the hydrophilic macromolecules dextran and Ficoll to study the effect of crowding in relative absence of hydrophobic interactions. In Supporting Information, Section 3, dextran (m. w. 6 000 g/mol) and Ficoll (m. w. 70 000 g/mol) were used as negative controls to show that these polymers do not selectively accelerate mismatched strand exchange over the baseline rate in pure buffer. Therefore, pure molecular crowding can be excluded as a mechanism.

## 5 CONCLUSIONS AND OUTLOOK

In conclusion, we have shown that a mismatched duplex is converted more readily into a matched duplex through strand exchange if PEG is present. Furthermore, the difference in exchange rates between a matched duplex and a mismatched duplex increases with PEG concentration, which means that a crowded and hydrophobic environment is important for the specificity of sequence recognition of DNA. These conclusions point in a direction that potentially contains the heart of mechanistic function of DNA strand recombinases.

It is getting increasingly clear that hydrogen bonds and base pairing alone do not decisively govern the stability of double stranded DNA. Instead, hydrophobic and dispersive interactions promoting base stacking are of predominant importance. Enzymatic DNA strand exchange is fundamental for the repair of DNA mismatches and is *in vivo* catalyzed by recombinases such as RecA and Rad51. These enzymes form elongated helical DNA‐protein complexes in which several DNA strands are surrounded by protein monomers.^[^ ^25^, ^26^, ^27^, ^28^, ^29^, ^30^ ^]^ Despite intense structural and functional studies, the strand exchange mechanisms that these recombinases mediate have remained largely unresolved.^[^ ^30^, ^31^, ^32^ ^]^ However, hydrophobic DNA‐protein interactions and DNA helix destabilization are two factors that have been considered important.^[^ ^33^, ^34^, ^35^ ^]^

Another perspective of the importance of high‐fidelity (thermodynamic as well as kinetic) DNA base recognition is for the formation of large unrepeated DNA nanoconstructs, in which all DNA sequences must be unique. The yields of such constructs become notoriously bad with a larger number of DNA strands or more complex designs,^[^ ^36^, ^37^ ^]^ which at least partially is due to the inability of a particular strand to avoid binding at an incorrect position. As a result, much of the original DNA is wasted on mismatched byproducts.^[^ ^38^, ^39^ ^]^ Understanding how mismatched aggregates can be resolved through strand exchange could potentially revitalize self‐building and addressable DNA nanotechnology.

## CONFLICT OF INTEREST

The authors declare no competing interests.

## Supporting information


**Appendix** **S1**: Supporting information

## Data Availability

The data that supports the findings of this study (DNA sequences, fitting details of kinetic traces, and melting curves) are available in the supplementary material of this article.
