# Supplementary material for: Mismatch detection in homologous strand exchange amplified by hydrophobic effects
Source: Biopolymers. 2021 Mar 29;112(4):e23426. doi: 10.1002/bip.23426 (PMC11475334; doi:10.1002/bip.23426)
Supplement: Supplementary file 1 — Appendix S1: Supporting information [file BIP-112-e23426-s001.pdf]

Supporting Information

Mismatch detection in homologous strand exchange amplified by hydrophobic effects

Bengt Nordén, Tom Brown, and Bobo Feng

Contents

Section 1. DNA sequences (p. 2)

Section 2. Curve fitting (p. 3)

Section 3. Negative controls (p. 11)

Section 4. Melting temperatures/curves (p. 12)

Section 1. DNA sequences

FAM-modified strand:	3’-GCA GTT GTA TGT ATA GTG GT-5’-FAM
Matching TAMRA-modified strand:	5’-CGT CAA CAT ACA TAT CAC CA-3’-TAMRA
Unlabeled strand (added 5 times in excess):	5’-CGT CAA CAT ACA TAT CAC CA-3’
Mismatched strand at position 4 (m4):	5’-CGT AAA CAT ACA TAT CAC CA-3’-TAMRA
Mismatched strand at position 5 (m5):	5’-CGT CCA CAT ACA TAT CAC CA-3’-TAMRA
Mismatched strand at position 10 (m10):	5’-CGT CAA CAT CCA TAT CAC CA-3’-TAMRA

Section 2. Curve fitting

The fluorescence kinetic traces were normalized to a yield between 0 and 1, using the initial fluorescence intensity before addition of the unlabeled strand, and the final fluorescence intensity. If the reaction is unfinished after 200 minutes, the (approximate) intensity corresponding to infinite time is obtained by heating the three strand DNA mixture well above melting temperature and re-annealing. By optimizing least squares of the error, the single exponential model $y = 1 - A \cdot \exp(-k \cdot x)$ was fitted to the experimental data. In the figures below, the horizontal axis is time (minutes), and the vertical axis is normalized yield.

0% PEG

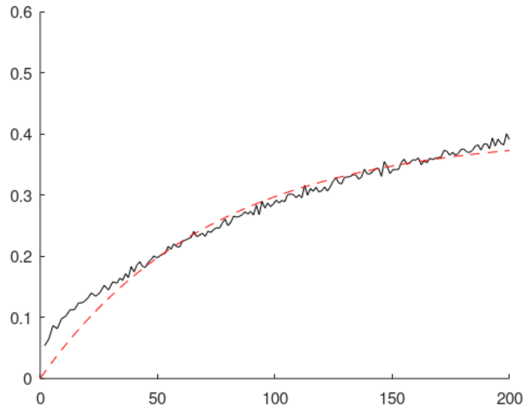

Matching A=0.398690 k=0.013703

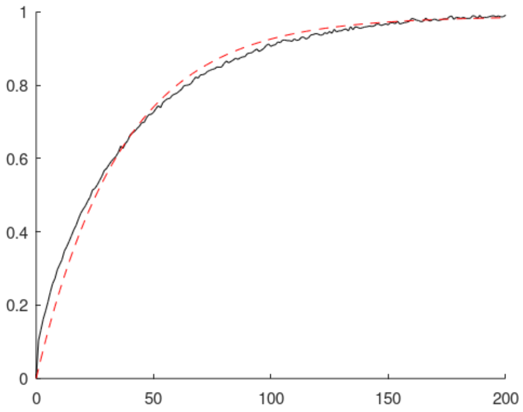

m4 A=0.987448 k=0.027683

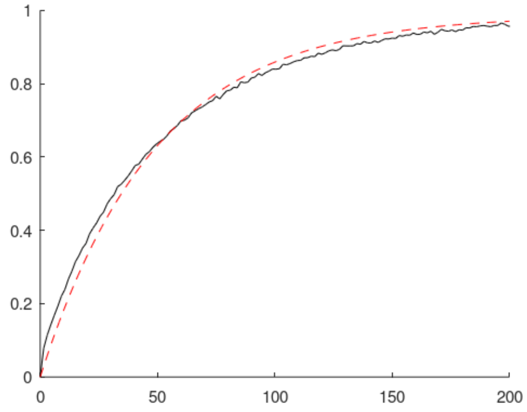

m5 A=0.986594 k=0.020417

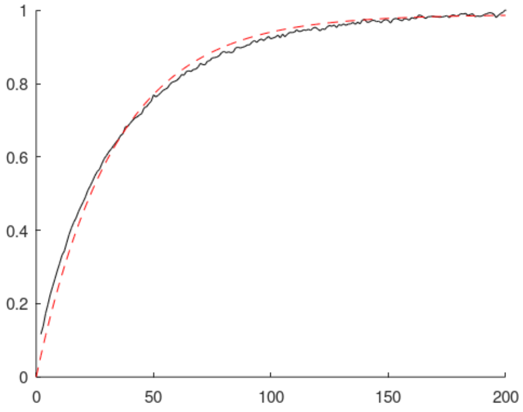

m10 A=0.988270 k=0.030252

30% PEG

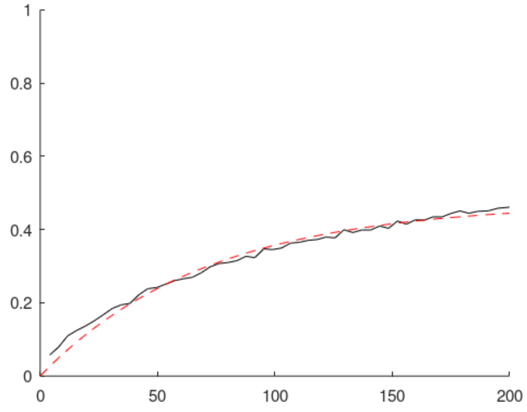

Matching A=0.472713 k=0.014094

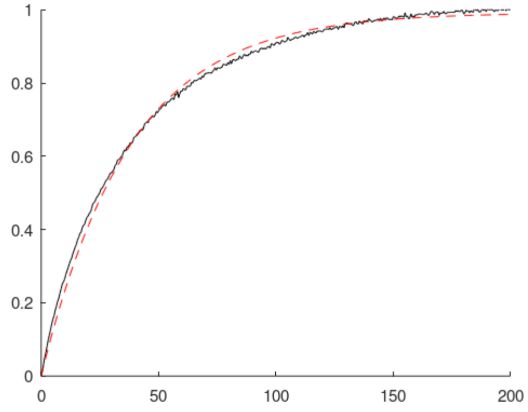

m4 A=0.992269 k=0.026557

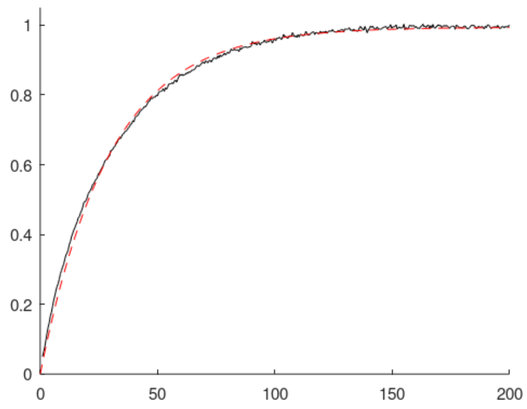

m5 A=0.994155 k=0.033965

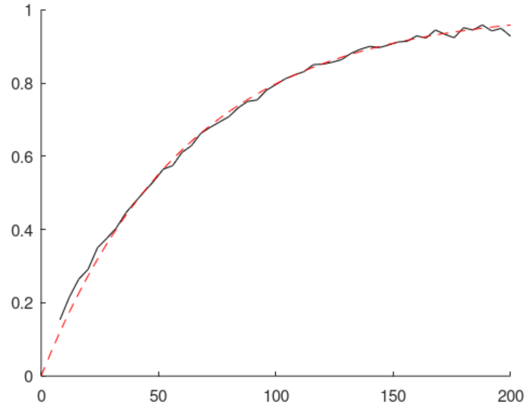

m10 A=0.998503 k=0.016032

40 % PEG

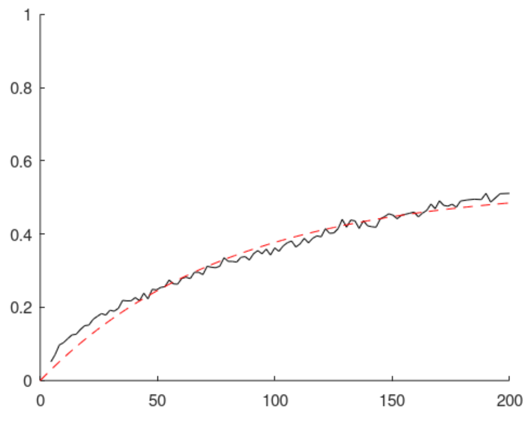

Matching A=0.527757 k=0.012537

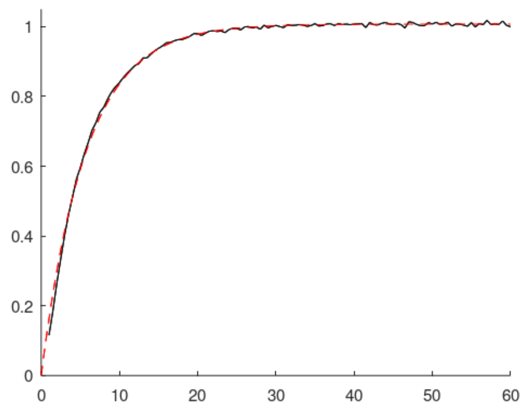

m4 A=1.0074 k=0.1769

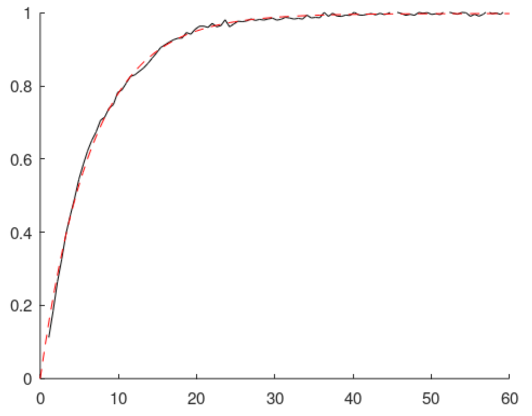

m5 A=0.9976 k=0.1523

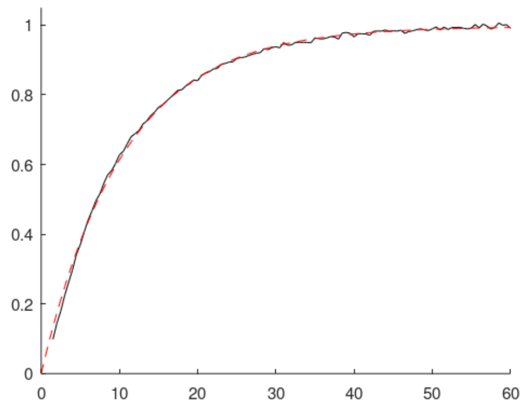

m10 A=0.996307 k=0.095627

45 % PEG

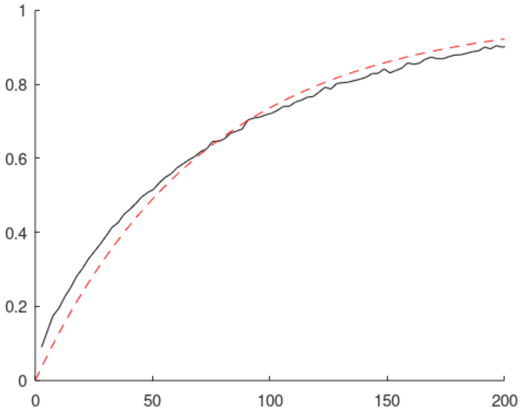

Matching A=0.984626 k=0.013765

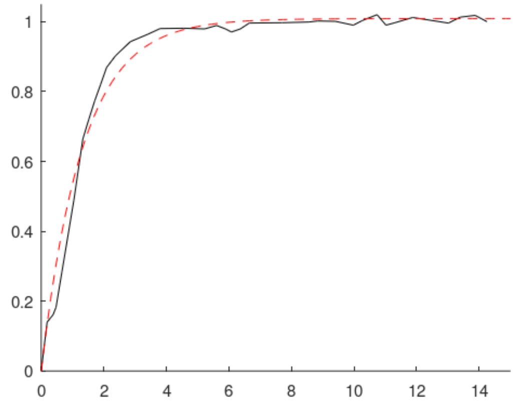

m4 A=1.0090 k=0.7590

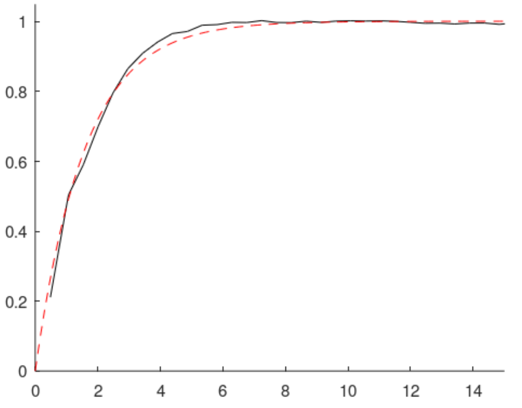

m5 A=1.0012 k=0.6354

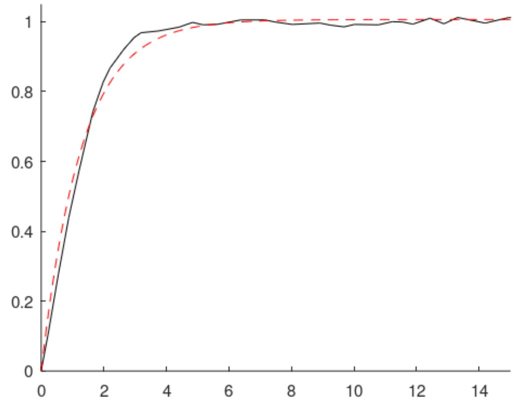

m10 A=1.0062 k=0.7816

0% PEG + salt

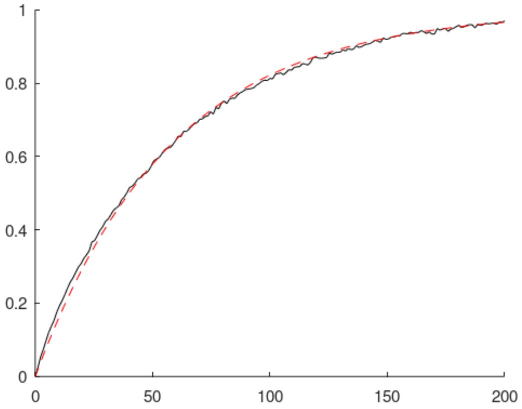

m4 A=0.996506 k=0.017400

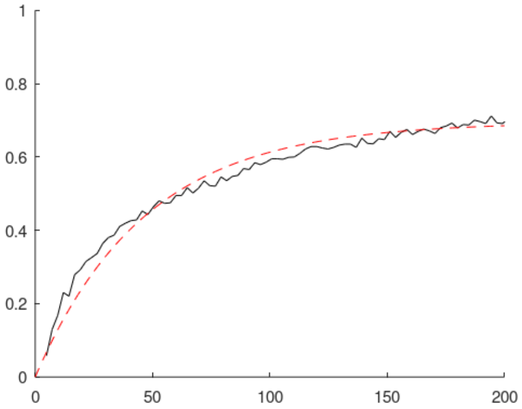

m5 A=0.694599 k=0.021398

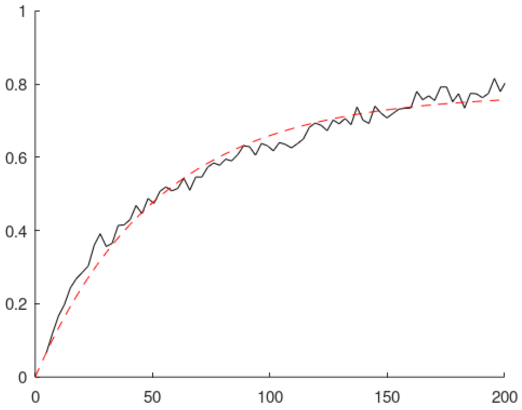

m10 A=0.773371 k=0.019121

30% PEG + salt

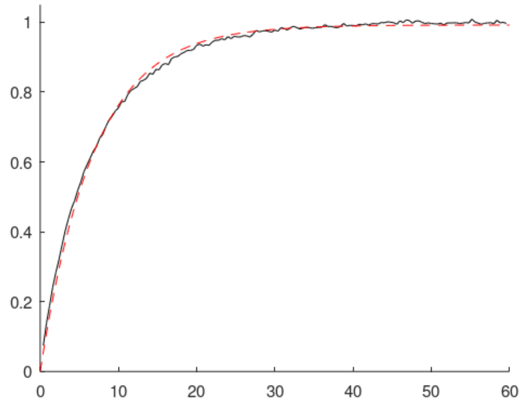

m4 A=0.9919 k=0.1463

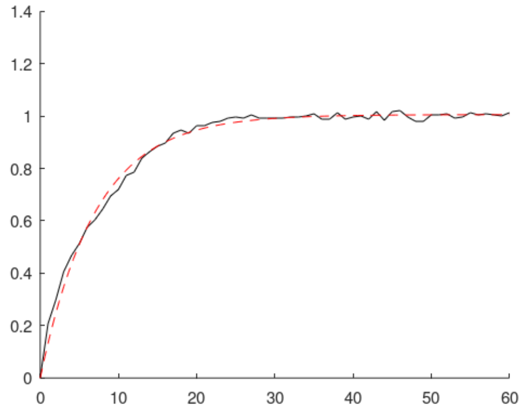

m5 A=1.0054 k=0.1416

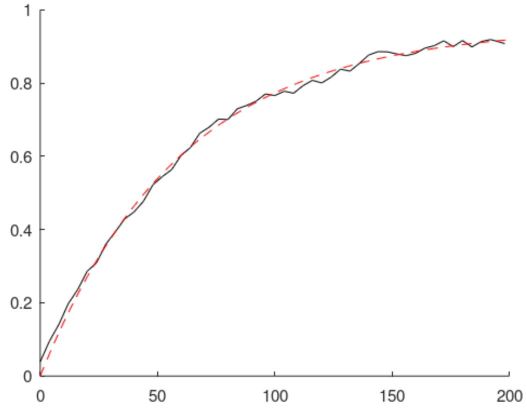

m10 A=0.951645 k=0.016717

40% PEG + salt

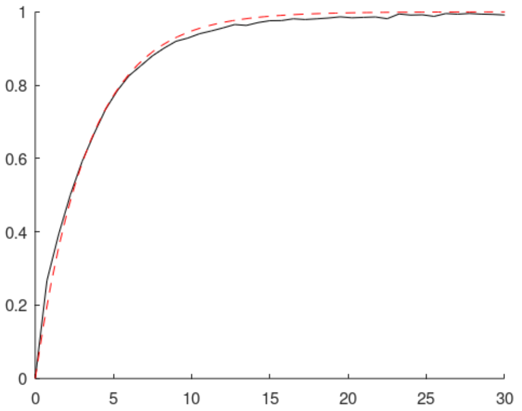

m4 A=0.9996 k=0.2955

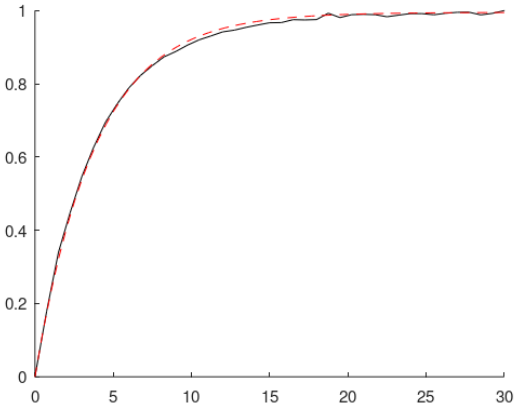

m5 A=0.9948 k=0.2600

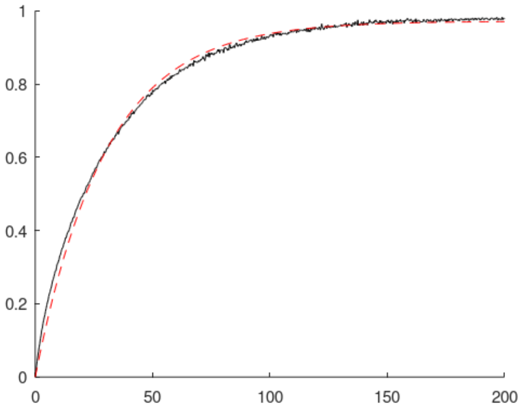

m10 A=0.971410 k=0.033504

45% PEG + salt

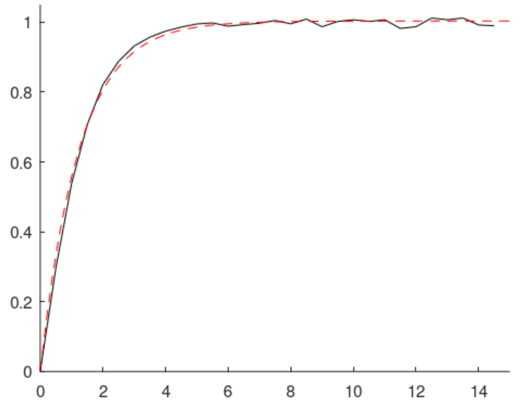

m4 A=1.0033 k=0.8113

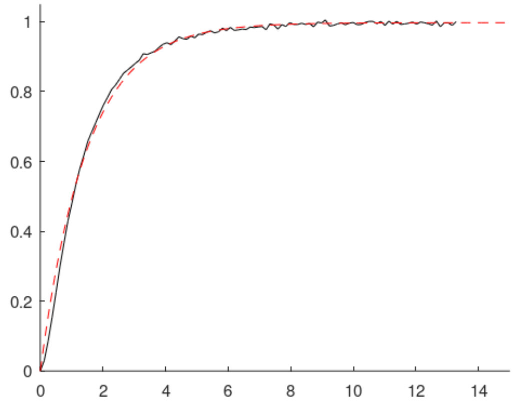

m5 A=0.9971 k=0.6784

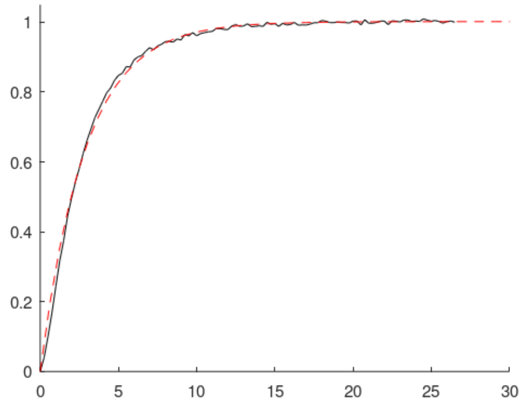

m10 A=1.0017 k=0.3502

Section 3. Negative controls

To verify that the increase in fluorescence is only due to strand exchange upon addition of the third unlabeled strand, negative controls were performed where an unrelated 18-mer DNA was added (indicated by arrows) instead of the complementary unlabeled strand. Blue lines show the raw FAM fluorescence intensity. The red dotted lines indicate the (hypothetical) fluorescence intensity at infinite time.

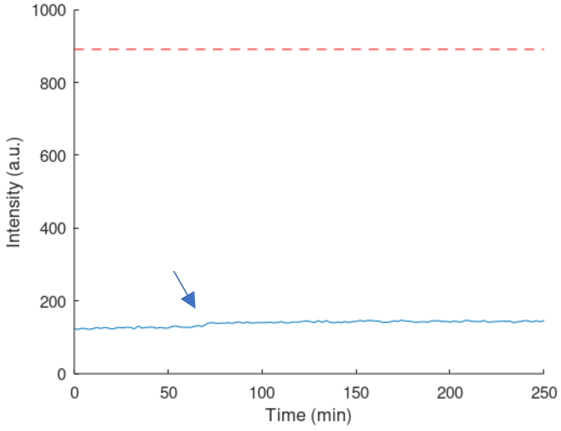

m4, 0% PEG

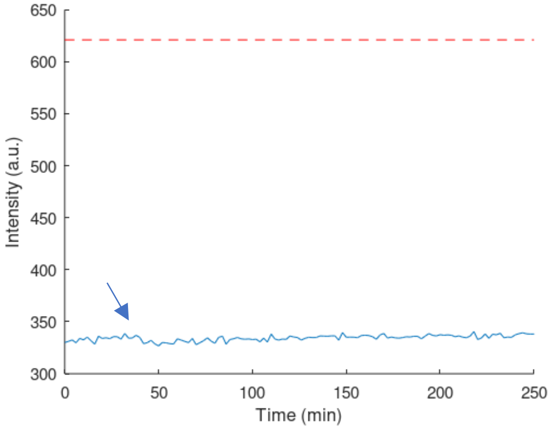

m4, 45% PEG

To exclude that the strand exchange accelerating effect of PEG 6000 is due to pure molecular crowding, control, control experiments were performed in 40% Dextran (MW=6000) and 40% Ficoll (MW=70’000). Mismatch strand exchange in these crowding but non-hydrophobic polymers is not significantly faster than in pure buffer.

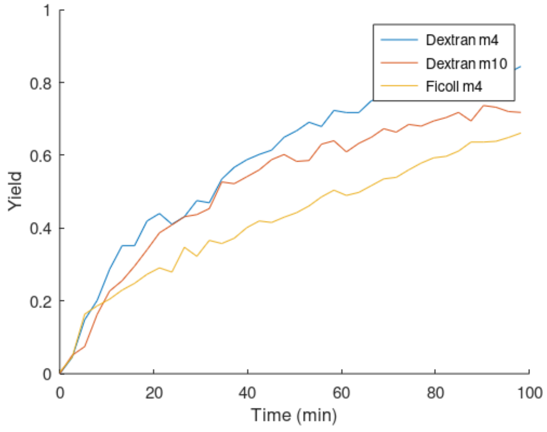

Section 4. Melting temperatures/curves

To compensate for the loss in duplex stability due to the mismatch, extra sodium chloride was added to the buffer before mixing with PEG to increase the melting temperature. The following table shows the melting temperatures (as obtained through fluorescence melting curves) and buffer salt concentrations of the various duplexes. The uncertainty in melting temperature is approximately ± 1 °C.

	Duplexes without extra salt ([Na^+] = 50 mM)				Duplexes with extra salt		
PEG concentration	Matched duplex	m4	m5	m10	m4	m5	m10
0 %	51 °C	45 °C	46 °C	45 °C	51 °C<br>140 mM	51 °C<br>140 mM	51 °C<br>140 mM
30 %	54 °C	47 °C	47 °C	47 °C	54 °C<br>180 mM	54 °C<br>180 mM	55 °C<br>180 mM
40 %	49 °C	42 °C	42 °C	43 °C	49 °C<br>180 mM	50 °C<br>180 mM	50 °C<br>180 mM
45 %	45 °C	39 °C	38 °C	40 °C	45 °C<br>200 mM	45 °C<br>200 mM	45 °C<br>180 mM

The fluorescence melting curves below correspond to the temperatures in above table. They were obtained by ramping at ~ 0.5 °C/min, although with the inherent limitation of the experimental setup that the recorded temperature refers to the thermostat block and not the contents of the quartz cells. Fluorescence increases upon duplex melting due to separation of the FRET pair. Fluorescence intensity is plotted black solid, its derivative (moving average with window size 5) black dotted, and the melting temperature (taken as the maximum derivative) red dotted. Dotted lines are for illustration only.

Melting curves for the first column:

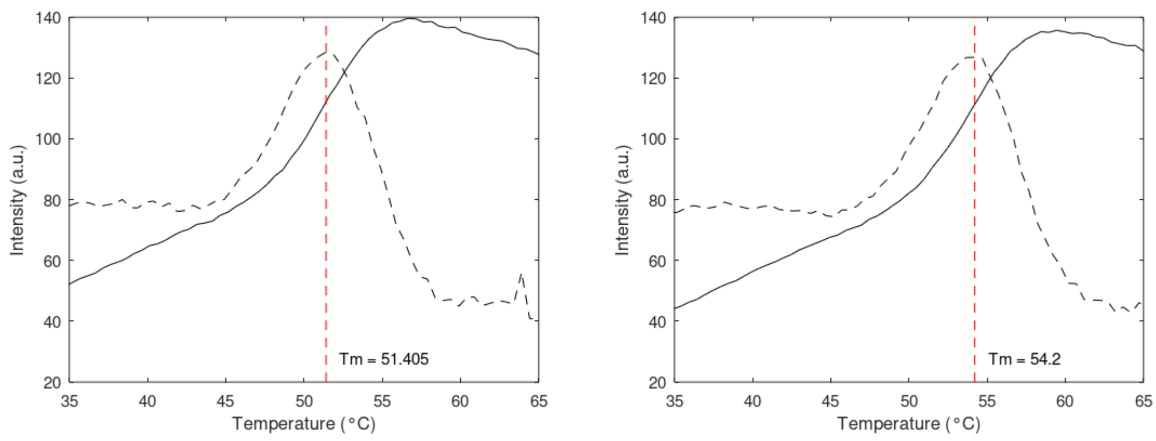

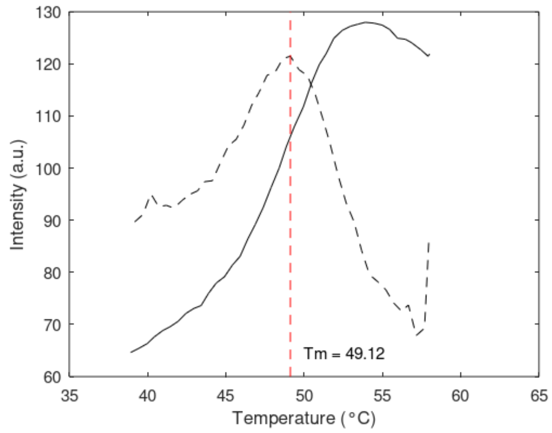

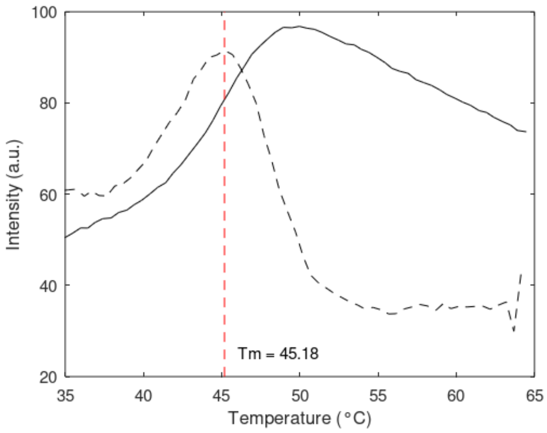

Melting curves for the second column:

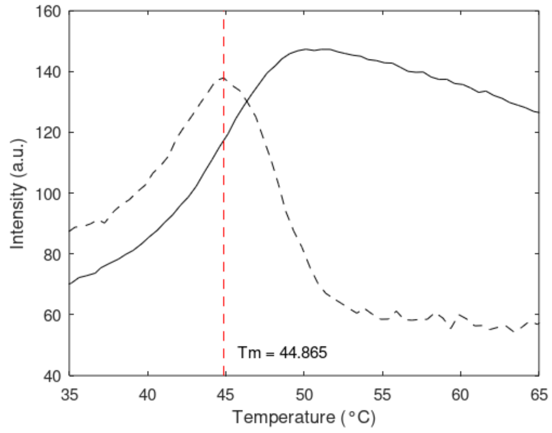

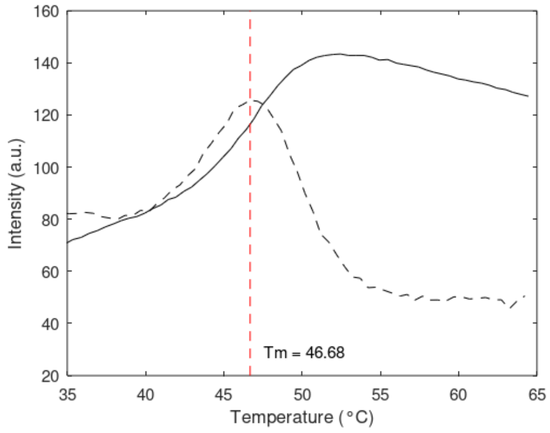

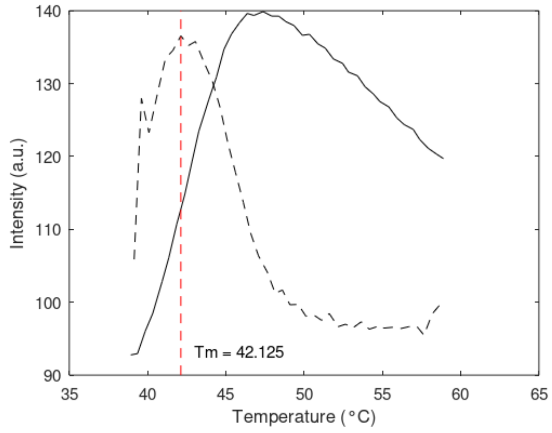

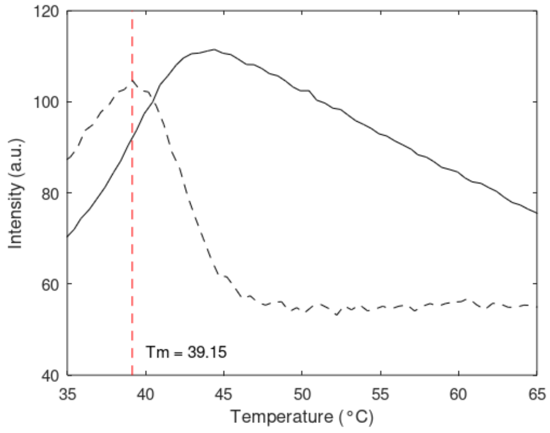

Melting curves for the third column:

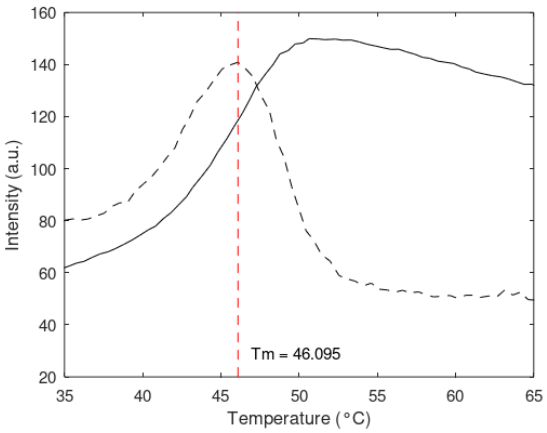

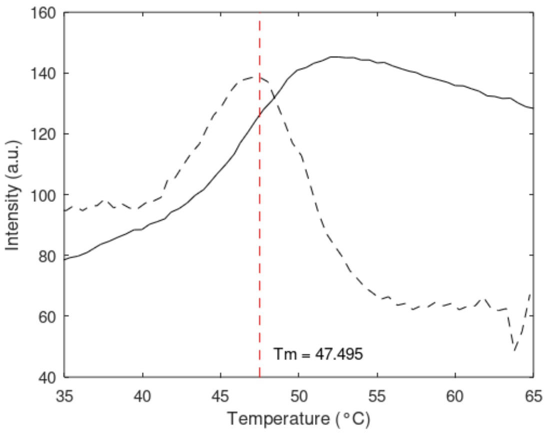

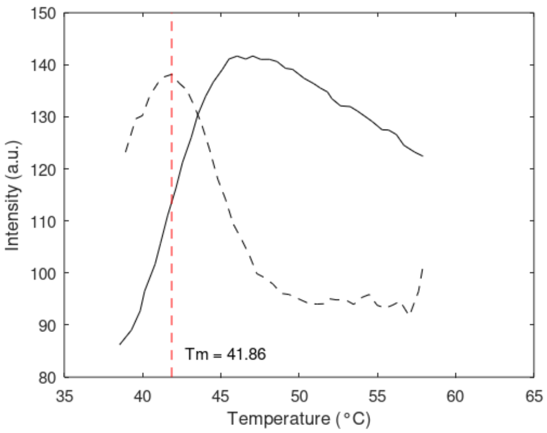

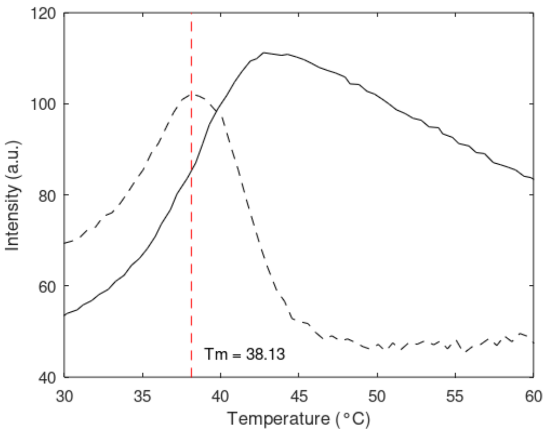

Melting curves for the fourth column:

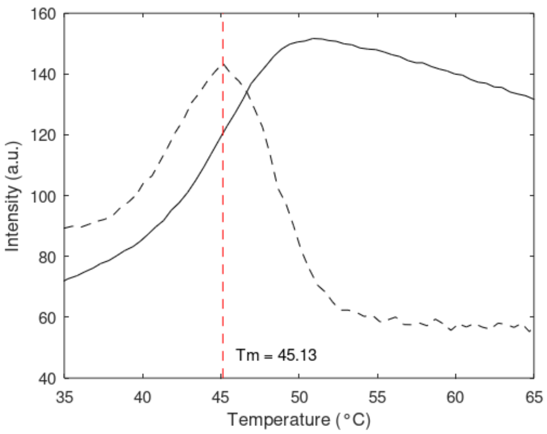

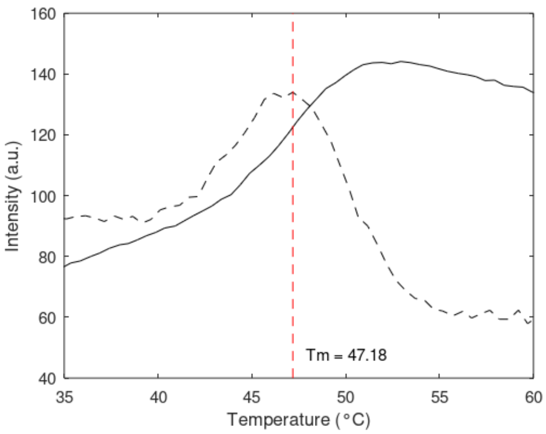

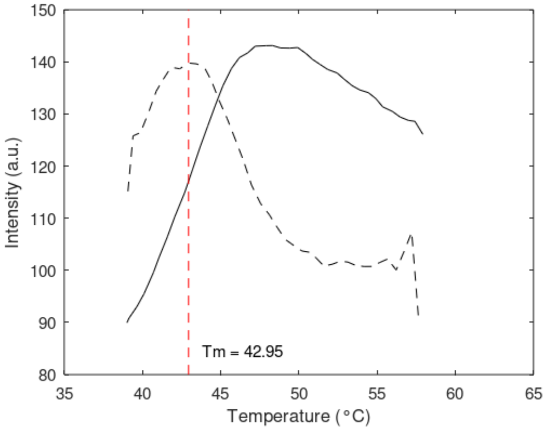

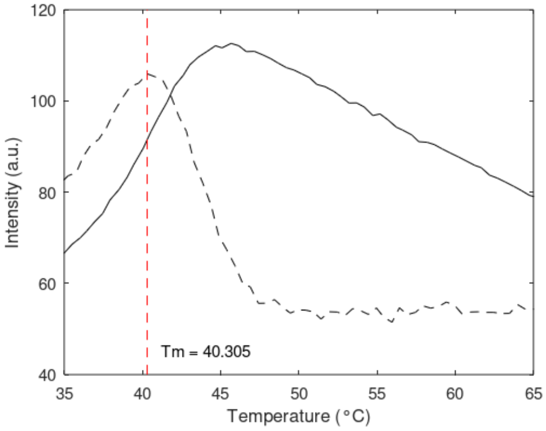

Melting curves for the fifth column:

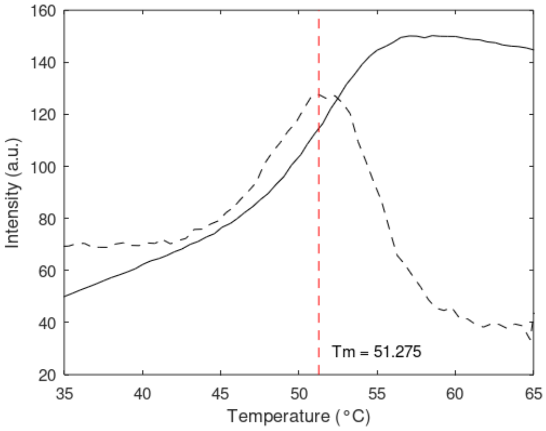

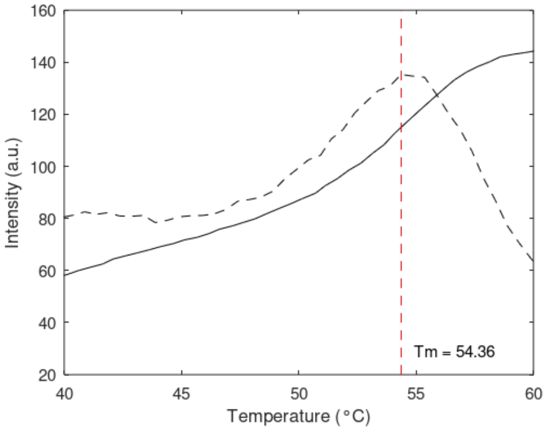

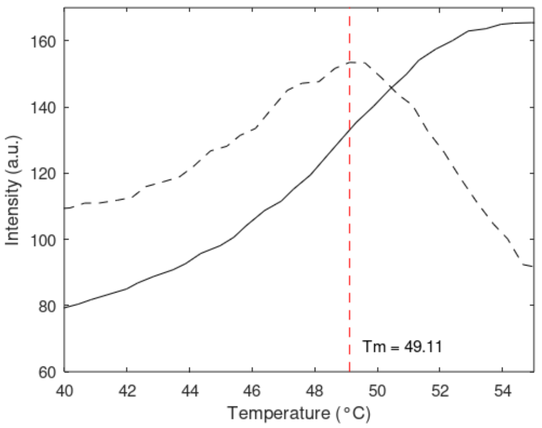

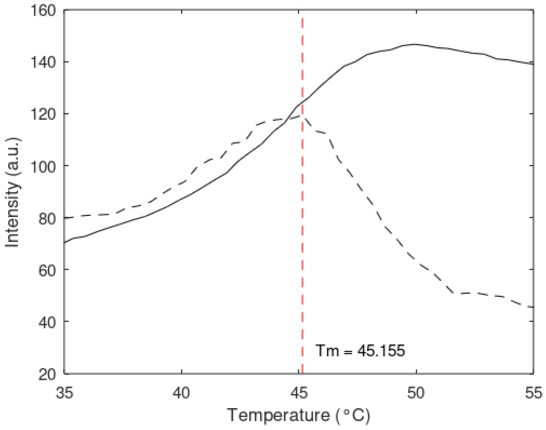

Melting curves for the sixth column:

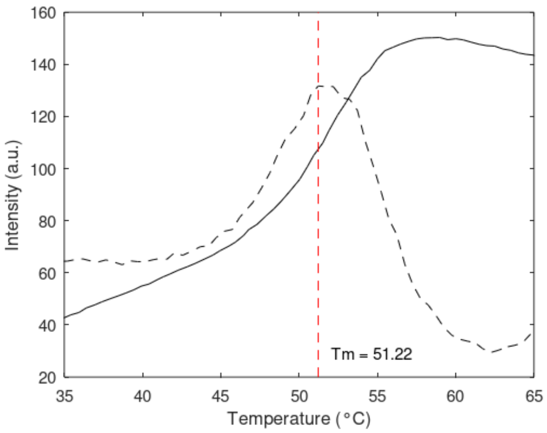

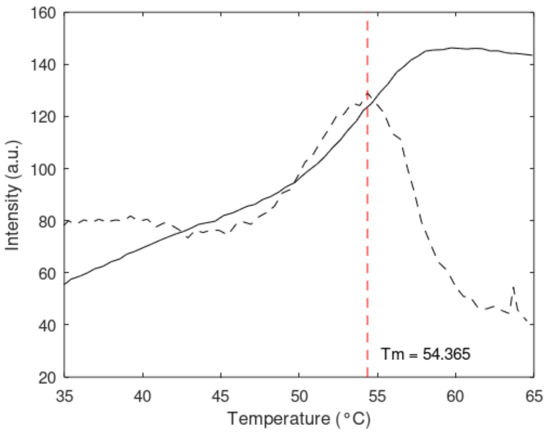

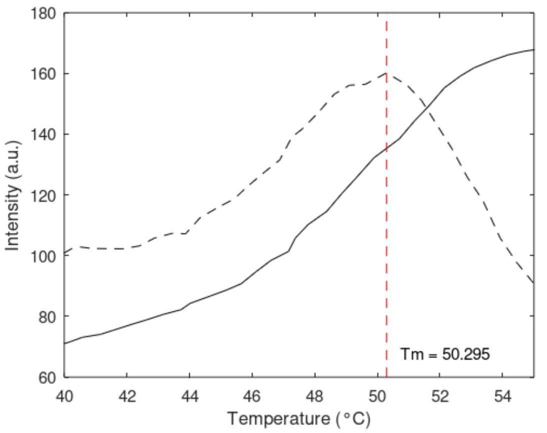

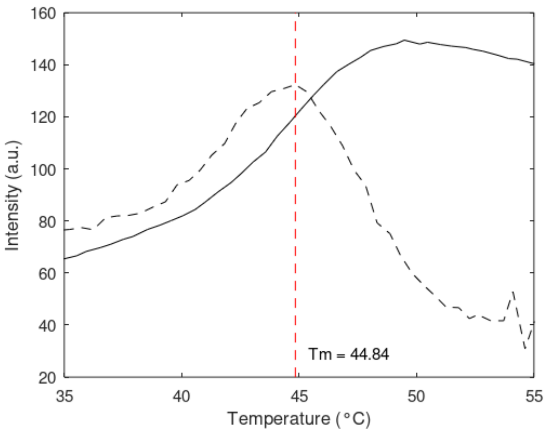

Melting curves for the seventh column:

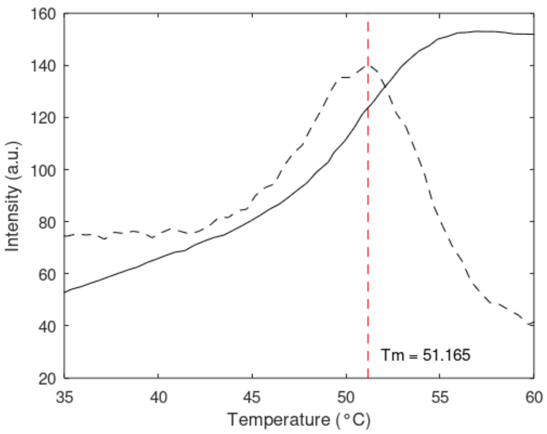

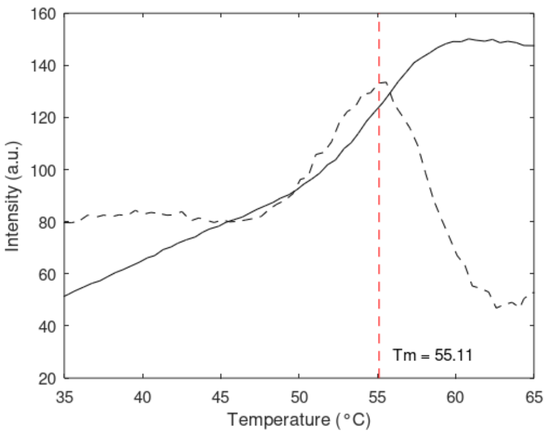

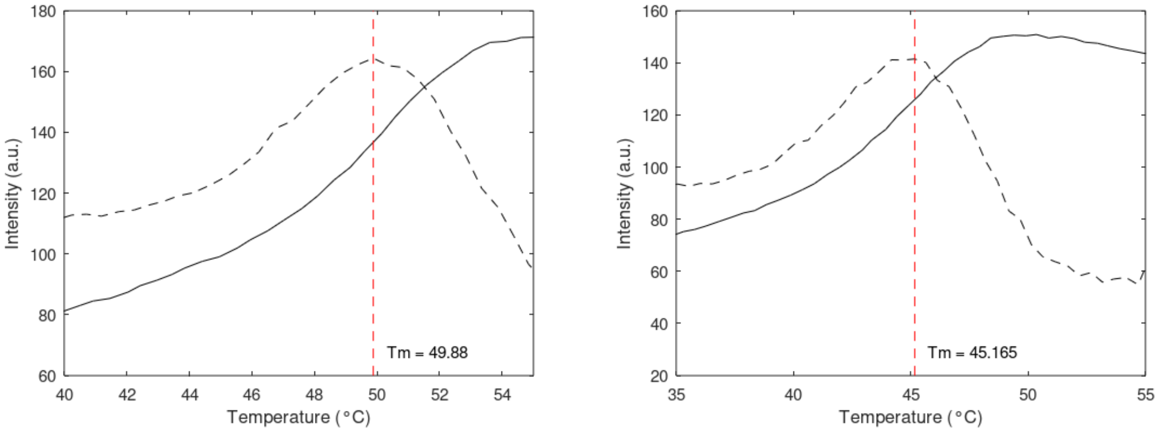
Intensity (a.u.)
Temperature (°C)
Tm = 49.88
Intensity (a.u.)
Temperature (°C)
Tm = 45.165
